# A New Global Mangrove Height Map with a 12 meter spatial resolution

**DOI:** 10.1038/s41597-024-04213-z

**Published:** 2025-01-04

**Authors:** Marc Simard, Lola Fatoyinbo, Nathan M. Thomas, Atticus E. Stovall, Adriana Parra, Abigail Barenblitt, Pete Bunting, Irena Hajnsek

**Affiliations:** 1 Jet Propulsion Laboratory, California Institute of Technology, Pasadena, USA; 2 NASA Goddard Space Flight Center, Biospheric Sciences Laboratory, Greenbelt, Maryland USA; 3 History, Geography and Social Sciences, Edge Hill University, Ormskirk, England; 4 University of Maryland, Earth System Science Interdisciplinary Center, College Park, Maryland USA; 5 Aberystwyth University, Aberystwyth, Wales UK; 6 ETH Zürich, Institut für Umweltingenieurwissenschaften, Zürich, Switzerland; 7 Microwaves and Radar Institute, German Aerospace Center, Cologne, Germany

**Keywords:** Ecosystem services, Marine biology

## Abstract

Mangrove forests thrive along global tropical coasts, acting as a barrier that protects coastlines against storm surges and as nurseries for an entire food web. They are also known for their high carbon sequestration rates and soil carbon stocks. We introduce a new global mangrove canopy height map generated from TanDEM-X spaceborne elevation measurements collected during the 2011–2013 period with a 12-meter spatial resolution and an accuracy of 2.4 meters (RMSE). Height was calibrated and validated using GEDI mission data and independently verified with airborne Lidar. The tallest mangrove stands reach nearly 60 meters in Colombia and Gabon, and potentially other countries. The map captures a broader range of canopy heights with finer spatial details than other available global products that use optical imagery. This new global mangrove height dataset can aid in evaluating mangrove ecosystem services at local and regional scales, improving our understanding of factors controlling mangrove structure, and supporting conservation, climate mitigation and adaptation strategies.

## Background & Summary

Mangrove forests thrive within the intertidal zones of tropical and subtropical regions. They exhibit distinct structural features attributable to a confluence of controls, including species composition, environmental conditions, and developmental stages. Mangrove forests provide a wide range of ecological services, including shoreline protection, carbon sequestration, water filtration, and habitat for numerous species of plants and animals^1,2^. Although mangrove forests account for less than 1% of the global forested area, they store a disproportionately large amount of carbon for their area, due to the combination of aboveground and belowground carbon stocks^3^. This total is estimated to be between 5.03 PgC and 11.7 Pg C, with 1.6PgC −1.75 PgC found in AGB alone^4,5^.

While regional rates of mangrove loss have decreased since 2000^6,7^, it is estimated that over 35% of mangrove forest area has been lost over the last century^8,9^ with the largest loss extents in Southeast Asia^9,10^. Given their important role as carbon sinks, habitat and for coastline protection, coupled with high rates of change, updated and multitemporal maps of mangrove characteristics are greatly needed.

Global spatially explicit data of vegetation structure, its variation and multitemporal changes are especially useful for carbon and other ecosystem service accounting, as well as biodiversity, hydrology and climate modeling^11^. A first global map of mangrove canopy height and aboveground biomass was generated for the year 2000 using spaceborne interferometric synthetic aperture radar (InSAR) elevation data from the Shuttle Radar Topography mission (SRTM) and the Ice, Cloud, and Land Elevation Satellite (ICESat) mission^4^, with a 30-m pixel spacing. In this paper we present a more recent global map of mangrove canopy height, with an updated mangrove extent, finer spatial resolution and vertical accuracy. We use elevation measurements from the TanDEM-X (**T**erraSAR-X **a**dd-o**n** for **D**igital **E**levation **M**easurement) mission^12^, canopy height measurements from the Global Ecosystem Dynamics Investigation (GEDI) instrument^13^, 2015 mangrove extent map from Global Mangrove Watch^6^ to generate the map, and use GEDI and Airborne Lidar Scanning (ALS) data for validation. This new map represents mangrove canopy height in the 2011–2013 period, which is when most TanDEM-X elevation measurements were acquired over mangroves. The map is thus more recent than the previous SRTM-derived mangrove height map^4^ and has a finer spatial resolution and better height accuracy. We find that our mangrove canopy height model achieves better or comparable performance relative to other state-of-the-art global and regional height products when compared to spaceborne, *in situ* or airborne measurements.

We anticipate that these updated estimates of mangrove height will be useful for management, policy and *in situ* studies, as well as improve quantitative evaluation and modeling of mangrove resilience and vulnerability^14^, Aboveground Biomass (AGB), ecosystem carbon stocks, emissions, and more^4,15,16^.

## Methods

### TanDEM-X DEM processing

We acquired a total of 1409 TanDEM-X Digital Elevation Model (DEM) geotiffs from the DLR Geoportal—https://eoweb.dlr.de/egp/— The data is accessible through a science proposal process with the German Aerospace Agency (DLR) (https://tandemx-science.dlr.de/cgi-bin/wcm.pl?page=TDM-Proposal-Submission-Procedure). Each file was 1° × 1° in geographic coordinates (WGS84) with a spatial resolution equivalent to 12 m. The TanDEM-X DEM vertical reference system is the WGS84 ellipsoid model. The TanDEM-X DEM in mangrove areas was generated with data acquired mostly between December 2010 and April 2013.

**Fig. 1:**
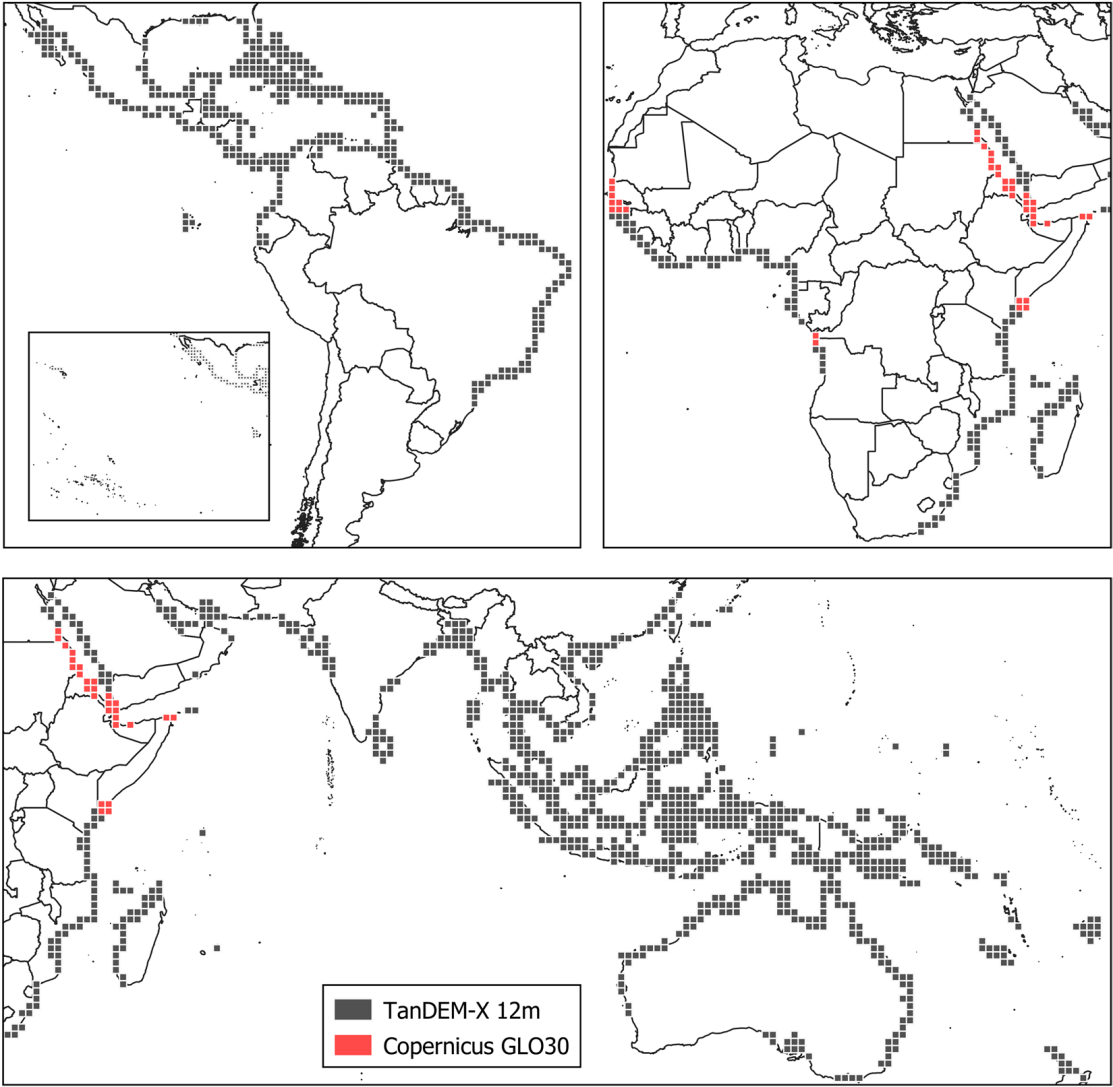
Distribution of 1° × 1^o^ DEM tiles. TanDEM-X 12 m and Copernicus GLO-30 DEMs tiles are in black and red respectively for the Americas (top left), West Africa (top right) and East Africa, Asia and Australia (bottom).

In areas where the 12-meters TanDEM-X data was not available, we completed the maps with the ESA Copernicus GLO-30 DEM^17^ (Fig. 1) (10.5069/G9028PQB). This DEM is a modified and updated version of the WorldDEM which is based on TanDEM-X DEM. GLO-30 has a spatial resolution of 30 m and is also distributed as 1^o^ × 1^o^ tiles. It has a horizontal coordinate reference system of geographic lat/lon (WGS84) and a vertical coordinate reference system of the Earth Gravitational Model 2008 (EGM2008). It is available globally with an absolute vertical accuracy better than 4 m. A total of 37 GLO-30 scenes (2.6%) were resampled to 12 meter using the nearest neighbor method to complete the TanDEM-X DEM. In addition, GLO-30 was downloaded for each TanDEM-X DEM tile for use in a noise reduction approach described below (Table 1).

**Table 1:** Parameters and thresholds for retaining high quality GEDI shots in the analysis.

Parameter	Threshold
Degraded flag	==0
Quality flag	==1
Number of detected modes	≧1 and <5
DEM elevation (from GEDI file) - EGM2008 geodetic elevation	<50 m
Landsat water persistence	<80
Absolute (Elevation of the lowest mode - mean sea surface)	<5
Total Energy	>2000 and <25000
Max TanDEM-X value	>0 m and <60 m
Min TanDEM-X value	<60
GEDI RH 98	>0 m and <60 m
TanDEM-X standard deviation. GEDI points with 3 to 4 pixels	<1.5 m
TanDEM-X standard deviation. GEDI points with 5 to 6 pixels	<2 m
TanDEM-X standard deviation. GEDI points with >6 pixels	<3 m

The TanDEM-X data comes with a water mask that is not detailed enough in mangrove systems where there are many narrow river channels and complex waterways. An additional water mask was therefore created using thresholding of 10 m Sentinel-1 SAR data in Google Earth Engine—https://developers.google.com/earth-engine/datasets/catalog/COPERNICUS_S1_GRD. For a given country, all Interferometric Wide (IW) swath mode Sentinel-1 images were collated for 2015 through 2016. For each image, a binary image was made using a backscatter threshold of −19 decibels. The images were then summed and each pixel total was divided by the number of pixels, providing a normalized water persistence image. Pixels with a normalized persistence value above 70% were classified as water.

To estimate canopy heights in mangroves, we subset the TanDEM-X DEM to the mangrove extent. We used Global Mangrove Watch (GMW) V3 (v3.14)^6^ which uses a combination of Landsat and Advanced Land Observing Satellite (ALOS) L-band Synthetic Aperture Radar (SAR) global mosaics for 11 epochs from 1996 to 2020 to create the first long-term time-series of global mangrove extent and change. To closely match the approximate date of the TanDEM-X data, we used the GMW 2015 baseline product. The GMW V3 has an estimated accuracy of 87.4% and it is expected that errors occur at the transitions from mangrove to upland tropical forests^6^. The GMW raster datasets are available (10.5281/zenodo.6894273) as 1^o^ × 1^o^ tiles in geographic coordinates (wgS84), with a spatial resolution of 25 m.

**Fig. 2:**
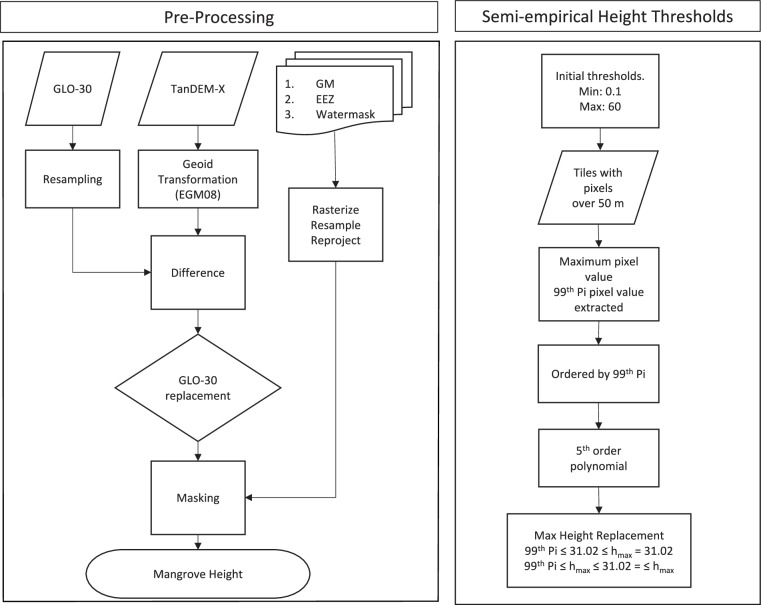
Diagram representing the processing steps for the Global TDX Mangrove Canopy Height creation.

The workflow shown in Fig. 2 operates on a per-tile basis and uses Exclusive Economic Zone (EEZ) information to assign pixels to a country. We distributed the data and processing across the NASA Advanced Data Analytics PlaTform (ADAPT). This is an efficient means of processing a global dataset, particularly for countries with large mangrove extents (e.g., Indonesia), in a consistent manner. Both raster and vector processing are based upon the Remote Sensing and GIS Library (RSGisLib) open source python software alongside a number of supporting modules and file formats including Pandas, GeoPandas, Numpy and GeoJSON.

**Fig. 3:**
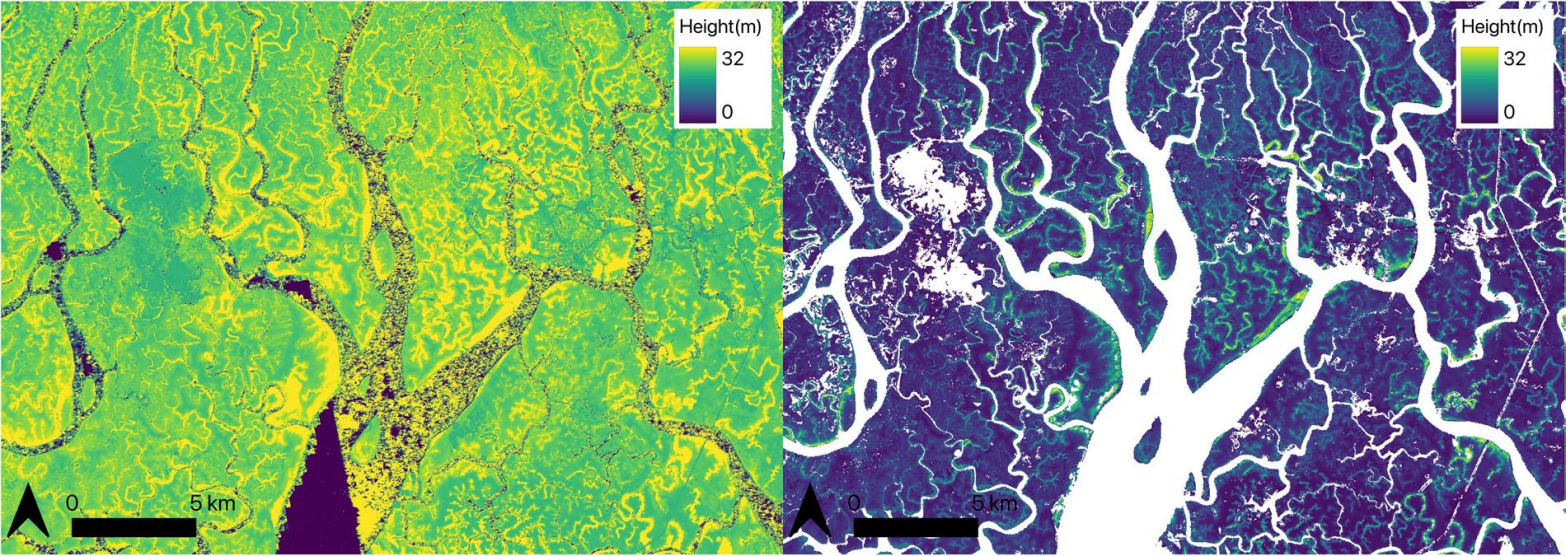
Masking and calibration of the TanDEM-X DEM. (**a**) TanDEM-X DEM before application of the water mask and (**b**) TanDEM-X canopy height map after water masking, subsetting to GMW and calibration to canopy height with GEDI Relative Height 98^th^ percentile (RH 98) data.

Initially, the auxiliary data including the EEZ extents, GMW and water mask were rasterized where necessary and subset to the TanDEM-X resolution, projection and grid, ensuring all TanDEM-X tiles had a suite of matching auxiliary data. Some TanDEM-X tiles contained image artifacts that were identified and replaced. To detect artifacts, the TanDEM-X DEM was converted from its vertical reference of WGS84 to EGM2008 to match that of the GLO-30 dataset, then each TanDEM-X tile was subtracted from its corresponding GLO-30 tile. Where the difference between the two datasets per-tile was greater than 30 m, the GLO-30 value was used. The noise-removed images were limited to a maximum height of 60 m, as pixel values outside of this were assumed to be above the maximum known mangrove height^4^. A minimum height of 0.1 m was also applied to remove ground pixels from the DEM. This resulted in 1446 EGM2008 TanDEM-X, including the 37 GLO-30 TanDEM-X-equivalent tiles (Fig. 1). Each DEM tile was then masked with the 2015 GMW baseline mangrove extent and Sentinel-1 derived water mask, resulting in 1446 mangrove-only DEM tiles (e.g. Fig. 3).

### Calibrating the TanDEM-X digital elevation model to mangrove canopy height

To calibrate the TanDEM-X DEM to mangrove canopy height, we used ancillary estimates of canopy height from NASA’s GEDI instrument. The GEDI instrument is a full waveform LiDAR system with a nominal footprint of ~25 m diameter that was launched to the International Space Station in 2018 to characterize forest vertical structure^13^. Using NASA’s Common Metadata Repository, we downloaded all GEDI files from the LP DAAC covering the GMW 2015 mangrove area that were publicly released up to May 2022, and extracted the relative height metrics available within the L2A product “Elevation and Height Metrics Data” version 002 — https://lpdaac.usgs.gov/products/gedi02_av002/. We only selected high quality GEDI data points, from here-on called ‘shots’, according to the quality flags established in the product’s guide that indicate non degraded or valid measurements^18^. Additionally, we established specific filtering parameters for our study based on expert knowledge of mangrove ecosystems. All filtering rules are shown in Table 1 and described below.

**Fig. 4:**
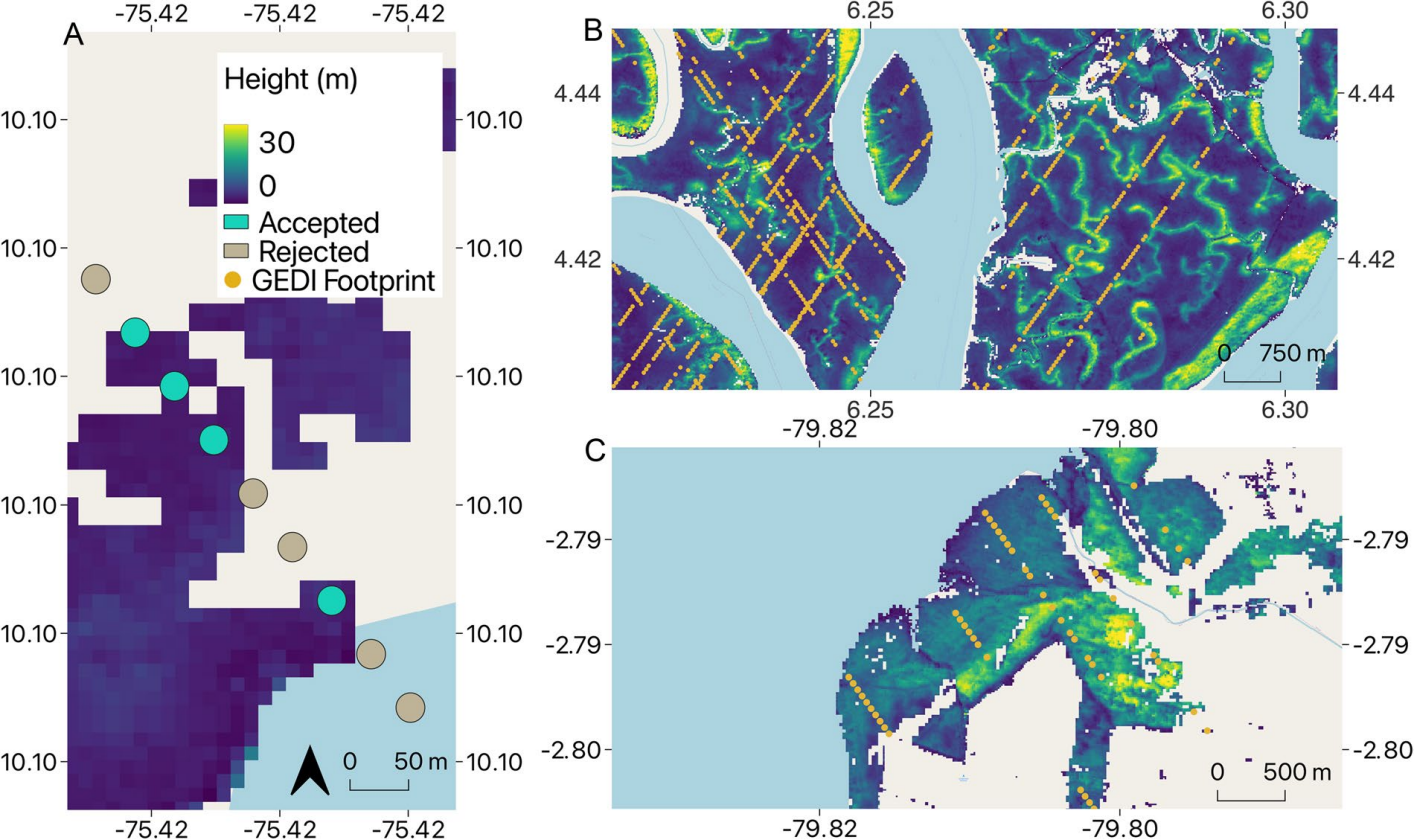
Example of GEDI shot coverage and selection. (**A**) Example of GEDI shot filters based on TanDEM-X coverage, with points in green shade retained in the analysis and GEDI shots in gray shade being removed from the analysis. Examples of selected GEDI shots coverage over the GMW 2015 area highlighting geographical variations in GEDI data availability for (**B**) the Niger delta in Nigeria, (**C**) the Guayas estuary in Ecuador.

The GEDI shots within the mangrove forests were transformed into 25 meters circular polygons and their Relative Height 98^th^ percentile (RH 98) was paired with the TanDEM-X DEM elevation values within each polygon. Additional filtering parameters were determined from analysis of these data. We removed GEDI shots based on the number of TanDEM-X pixels falling within each footprint polygon and their standard deviation (Table 1): First, we determined that GEDI shots covering less than three TanDEM-X pixels, which were commonly located at the edge of the GMW boundaries (Fig. 4), did not contain sufficient DEM information to confidently characterize the canopy structure observed by a GEDI shot. Thus, they were removed from the analysis. Secondly, we found that GEDI shots falling over mangrove areas with heterogeneous TanDEM-X height, resulted in larger discrepancies between the GEDI RH 98 and the maximum DEM value. These height discrepancies may be related to changes in canopy structure occurring between the acquisition dates of the two datasets, or to geolocation errors of the GEDI shots^19^. To reduce the impact of these factors, we developed a scheme that sets a maximum acceptable TanDEM-X height variation within a GEDI shot as a function of the number of TanDEM-X pixels within. That is, for GEDI shots with more than 6 TanDEM-X pixels, the standard deviation of the TanDEM-X values could not exceed 3 m while GEDI shots covering less than six DEM pixels were retained in the analysis if the standard deviation of the TanDEM-X pixels was below 1.5 m or 2 m in the number of pixels was in the ranges [3,4] and [5,6] respectively (see Table 1).

A final filtering step removes outliers by grouping the dataset into two-meter intervals of GEDI RH 98 and two-meter intervals of TanDEM-X DEM elevation and calculating the mean and standard deviation of the DEM values and RH 98 of each bin. We removed GEDI shots with RH 98 or TanDEM-X DEM values beyond three standard deviations from the mean in each bin. These additional filtering parameters provide safeguard against areas of land cover change, disturbances and significant tree growth that may have occurred in the 2011–2022 acquisition periods covered by TanDEM-X and GEDI.

The filtering process resulted in a final global dataset of 2 414 652 GEDI shots acquired between 2019-04-18 and 2022-05-22, with an average bias between GEDI RH 98 and TanDEM-X DEM elevation values of 1.7 m. To correct the bias, we constructed a calibration regression relating GEDI RH 98 to the maximum TanDEM-X DEM value. For this process we first divided the final global dataset into training and validation by dividing the GEDI RH 98 values into 6 height categories ([0, 10] m, [10–20], [20–30], [30–40], [40–50], $\ge$50) (See Table 2), and randomly selecting in each 70% (1,690,258) of the points for training and 30% (724,394) points for validation. To guarantee that all height categories were equally weighted in the regression model, we weighted each category according to their available number of points:

1 $${\rm{Weight}}=1/({\rm{\#of\; points}}\ast 6)$$

**Table 2:** Distribution of training shots per height category and weight values.

GEDI RH 98 bins	Number of points	Weight
0–10	1532815	0.0000001087
10–20	716754	0.000000232
20–30	144514	0.00000115
30–40	19830	0.0000084
40–50	684	0.000244
50–60	55	0.0030

To select the final calibration regression, we evaluated several models, including linear, power and quadratic regressions, and evaluated their fit based on the Pearson correlation coefficient (CORR), the mean absolute error (MAE), the root mean squared error (RMSE), and the bias. The best calibration equation, which corresponds to a linear regression with a square root transformation, is the following:

2 $${\rm{G}}{\rm{E}}{\rm{D}}{\rm{I}}\,{\rm{R}}{\rm{H}}98=({1.02\ast {\rm{s}}{\rm{q}}{\rm{r}}{\rm{t}}({\rm{T}}{\rm{D}}{\rm{X}})+0.33)}^{2}$$

**Fig. 5:**
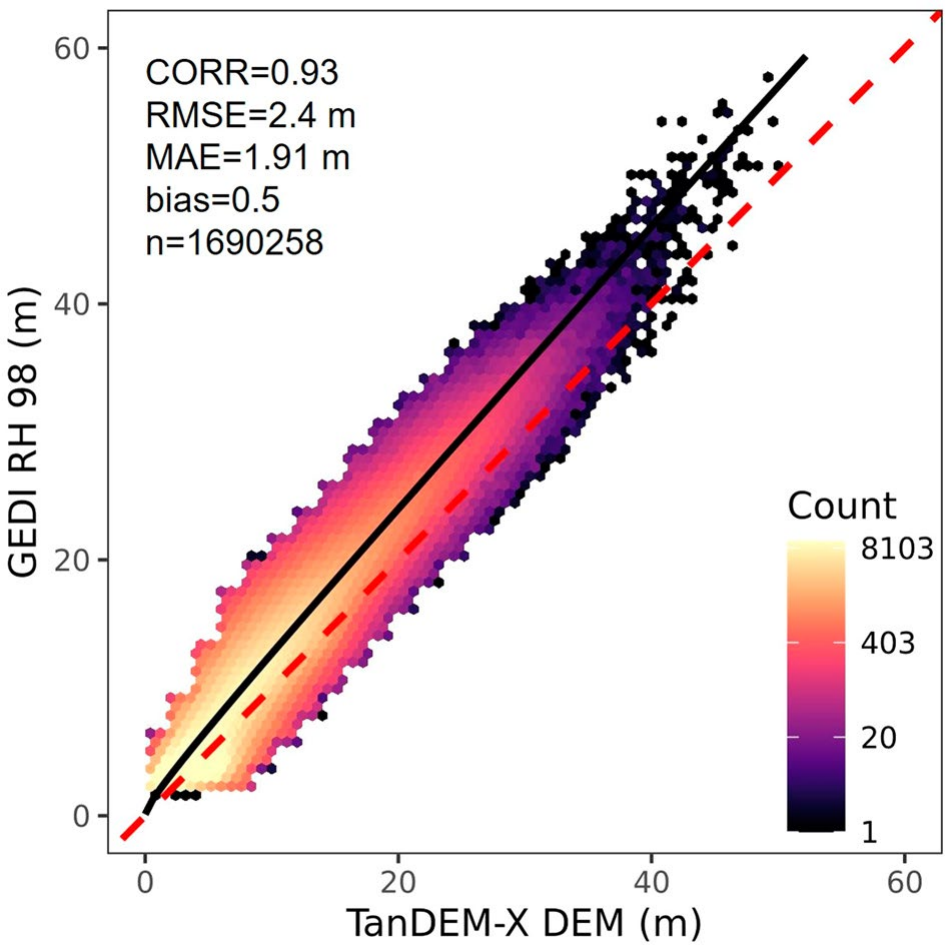
Canopy height regression used to generate TanDEM-X calibration with global GEDI RH98 data. The regression line is shown in black and the 1:1 is shown in red for reference.

Application of Eq. (2) to the TanDEM-X DEM values produces the calibrated mangrove canopy height as detected by the GEDI instrument (i.e. RH 98) with an RMSE and MAE of 2.4 m and 1.9 m respectively (Fig. 5).

### Post processing

**Fig. 6:**
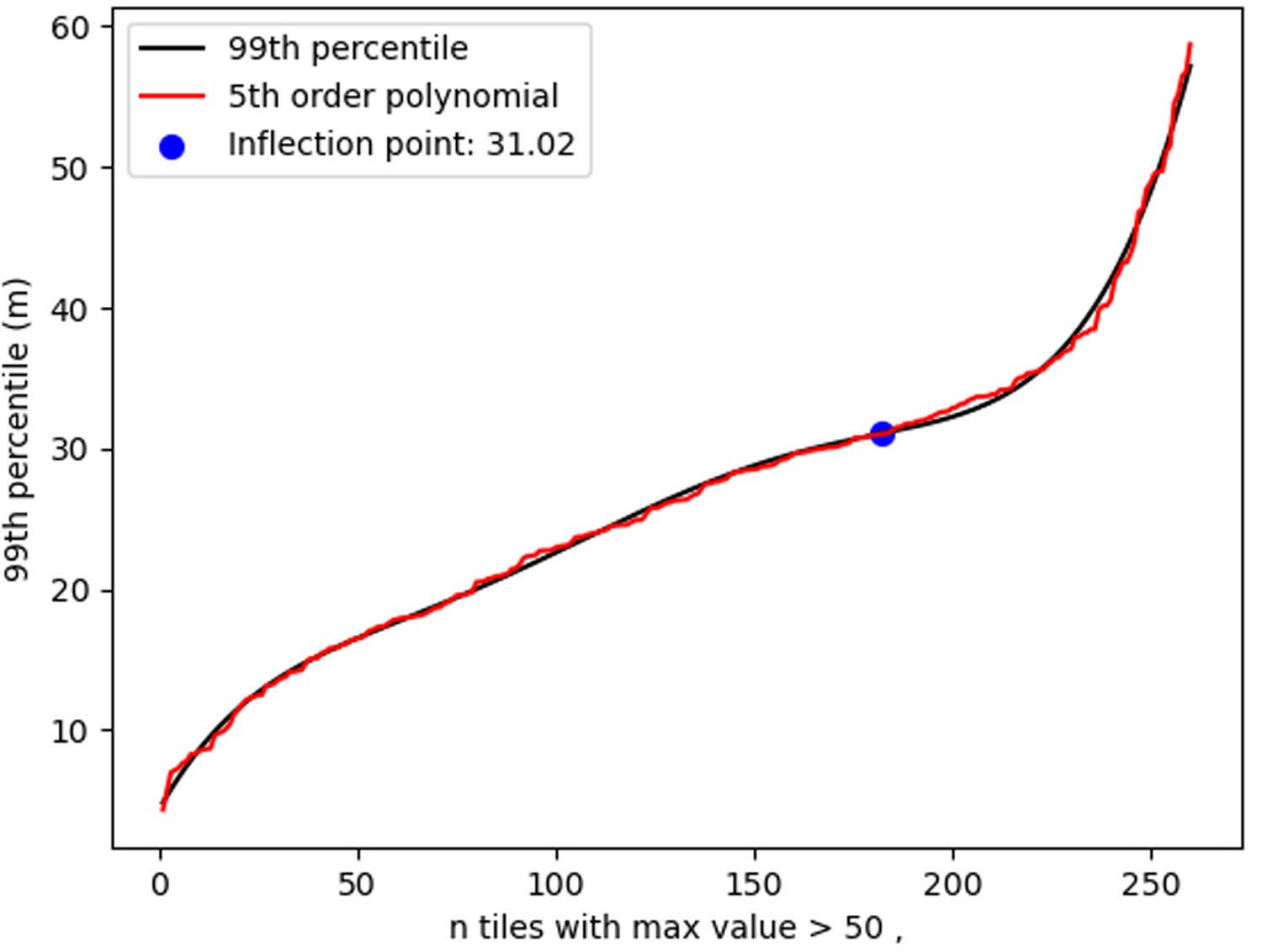
Mangrove height thresholding. Fifth-order polynomial regression of the 99th percentile DEM elevation of all tiles with elevation greater than 50. The inflection point is 31.02 meters.

GMW misclassification of inland forests and hills within the mangrove extent causes abnormally high mangrove height values in some locations. This is most often observed on islands with mangrove extents adjacent to steep mountainous terrain as well as dense broadleaf tropical forests adjoining mangrove forests. To mitigate their impact on national statistics while maintaining locations where tall (>55 m) mangrove stands may exist, we used a semi-empirical approach to identify and remove these outliers. All DEM tiles with a pixel value > 50 m were identified and the maximum and their respective 99th percentile value was calculated. These data were ordered by 99th percentile and a 5th order polynomial was fitted to the ordered data to locate the inflection point (Fig. 6), which occurs at 31.02 m. For all DEM tiles with a pixel value > 50 m, we then replaced the maximum elevation value by the 99th percentile if the 99th percentile value was less than 31.02 m. For tiles where the 99th percentile value > 31.02 m, the maximum elevation was unchanged.

## Data Records

**Fig. 7:**
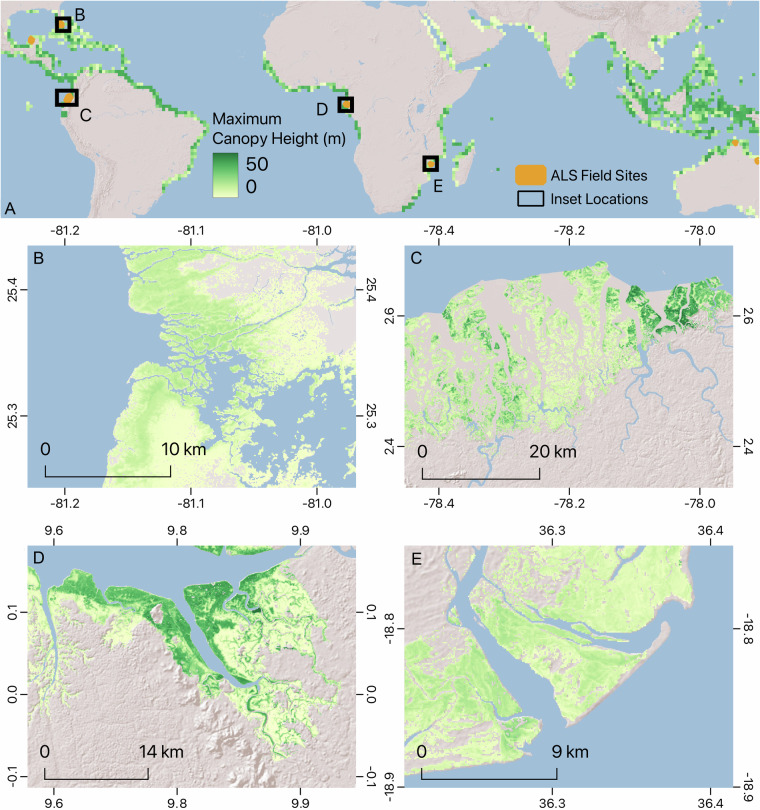
Global TDX Mangrove Canopy Height. (**A**) Green colors show the tallest mangrove canopy height found within 1° cells. The map also shows the locations of the Lidar sites and the locations of the high-resolution insets in b–e. (**B**) Everglades, Florida (USA). (**C**) Río Rosario (Ecuador). (**D**) Komo Estuary (Gabon). (**E**) Marromeu National Reserve (Mozambique).

The new global mangrove height dataset (Fig. 7) is available at ORNL DAAC^20^ (10.3334/ORNLDAAC/2251) and was generated using the TanDEM-X DEM calibrated with GEDI data collected between April 2019 and May 2022. The dataset can be downloaded on a country basis or 1 degree tiles. Each file is accessible as a Geotiff image wherein non-mangrove pixel values are set to zero “0”. The filtered GEDI data (Gedi_Mangrove_Height.csv) is also available at ORNL DAAC^20^. The data is formatted as a CSV file containing the geolocation, data quality, and canopy height parameters included in the L2A GEDI product, along with the extracted data from the TanDEM-X data, namely mean, minimum, maximum, standard deviation and pixel count. The data can also be visualized through a Google Earth Engine app at https://mangrovescience.earthengine.app/view/globalmangroveheighttdx.

## Technical Validation

We assess the quality of the dataset through analysis of the mangrove height statistics, comparison with existing datasets, and through validation with independent GEDI and airborne Lidar canopy height estimates.

### Country statistics

We first assess the overall realism of the dataset by identifying the countries with the tallest mangroves, associating each pixel with their respective country as specified by the EEZ lookup values. We selected the tallest 100 pixel values for each country. These were exported to a pixel file on a per-tile basis. In addition, the histogram of all mangrove DEM values was recorded on a per-tile for each country present in a tile. These files containing these histograms and the top 100 values were combined to generate country-level statistics. The median of the top 100 values per country and the 99th percentile were calculated. These values were written to an EEZ GeoPackage, thus providing a lookup table of mangrove canopy height values per-country. Mangrove height statistics were aggregated on a per-country basis. The top 20 countries with the tallest mangroves (by 99th percentile height) are provided in Table 3. This table highlights some issues related to errors in the GMW mangrove extent that include non-mangrove areas exhibiting unrealistic heights. For example, Japan and Palau do not have mangrove forests reaching over 56 m. Therefore, users should be wary of extreme values and investigate spatial evidence, particularly in island nations and proximity to hills and mountains.

**Table 3:** Top 20 countries with tallest mangrove canopy height.

Country	99th Percentile	Median of top 100 pixels
Colombia	46.1	59.6
Gabon	43.5	59.1
Equatorial Guinea	39.6	49.0
Panama	38.9	54.9
Costa Rica	38.4	51.9
Palau	37.6	59.5
Japan	37.4	56.6
Ecuador	37.2	57.4
Angola	37.0	55.7
Venezuela	36.4	59.1
French Guiana	35.2	49.5
El Salvador	34.9	46.0
Solomon Islands	32.4	59.7
Papua New Guinea	32.3	57.8
Cameroon	32.3	51.0
Indonesia	32.1	59.9
Democratic Republic of the Congo	32.0	45.5
Suriname	31.8	48.2
Guyana	31.7	41.0
Brazil	31.3	58.7

The table indicates some topographic artefacts remain in a few countries that most likely do not exhibit such tall mangrove forests.

### Validation with GEDI

**Fig. 8:**
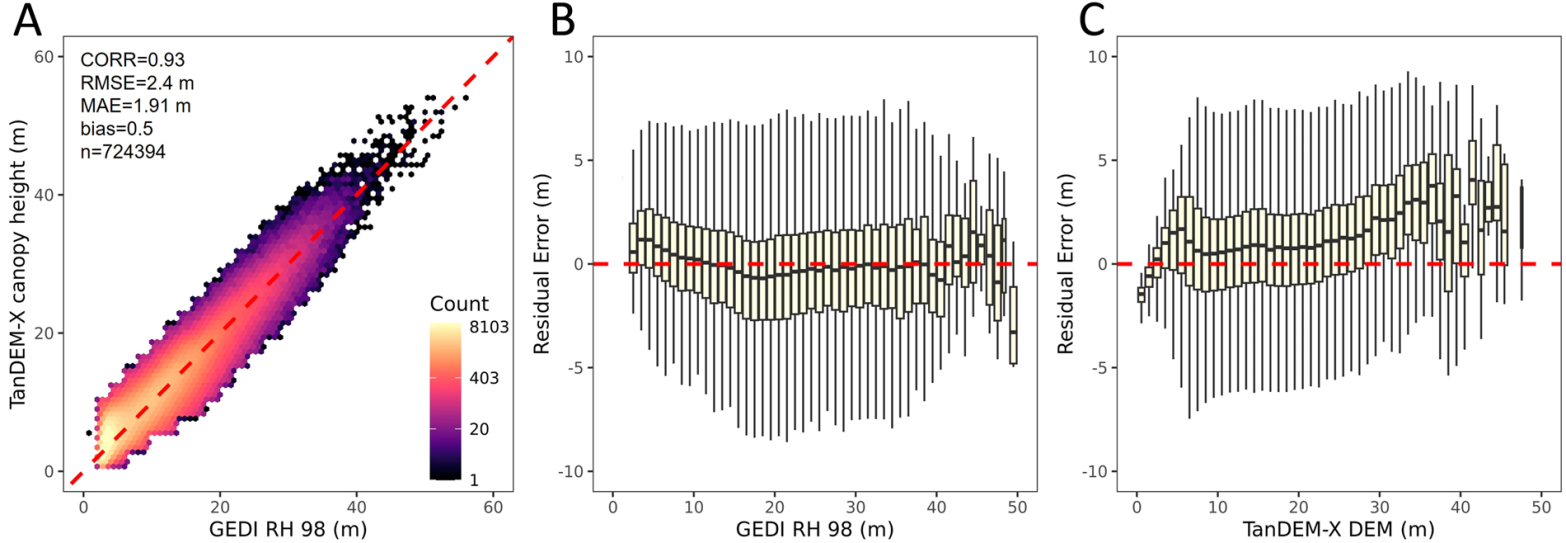
Validation of TDX mangrove canopy height (TMCH) estimates with globally distributed GEDI RH98. (**A**) Regression fit for the validation dataset, with resulting accuracy assessment statistics. The 1:1 line is shown as a dashed line for reference. Residual error (TMCH - GEDI RH98) distribution for the validation dataset as a function of (**B**) GEDI RH 98 values.

We used 70% of GEDI RH 98 measurements to derive a TanDEM-X calibration regression, and kept 30% of the samples for validation. Figure 8 shows the validation of the calibrated TanDEM-X mangrove canopy height with a bias of 0.5 m, an RMSE of 2.4 m, and a MAE of 1.91 m which is commensurate with the value obtained for the regression (Fig. 5). The variation of residual errors (TanDEM-X canopy height minus GEDI RH98) with canopy height measured by GEDI RH98 (Fig. 8B) indicates the TanDEM-X canopy height map slightly overestimates height of shorter canopies below 10 m. However, there are not enough samples in tall—greater than 40 m— to conclude on biases.

### Validation with airborne lidar

We used airborne laser scanning data (ALS) to validate the calibrated TanDEM-X mangrove canopy height map (Table 4). We used ALS data from 6 locations - Australia, Colombia, the United States, Mexico, Mozambique, and Puerto Rico. All ALS data was acquired within 5 years of the TanDEM-X measurement period.

**Table 4:** Airborne laser scanning data (ALS) used for validation of the mangrove canopy height.

Location	Date	Data Provide	Source	Availability	Link
Florida Eveglades, United States	2017	G-LiHT	NASA/Goddard Space Flight Center	Open	https://glihtdata.gsfc.nasa.gov/
Yucatan Peninsula	2013				
Puerto Rico	2017				
Zambezi River Delta, Mozambique	2014	Land Resources International	NASA CMS	Open	10.3334/ORNLDAAC/1521
Australia	2017	Airborne Research Australia	Auscover	Open	qld.auscover.org.au/public/data/mangroves/ARA_2017
Pacific Coast, Colombia	2018	Chemonics International and OPTIM	NASA/Jet Propulsion Laboratory	Private	10.1186/s13021-019-0117-9

**Fig. 9:**
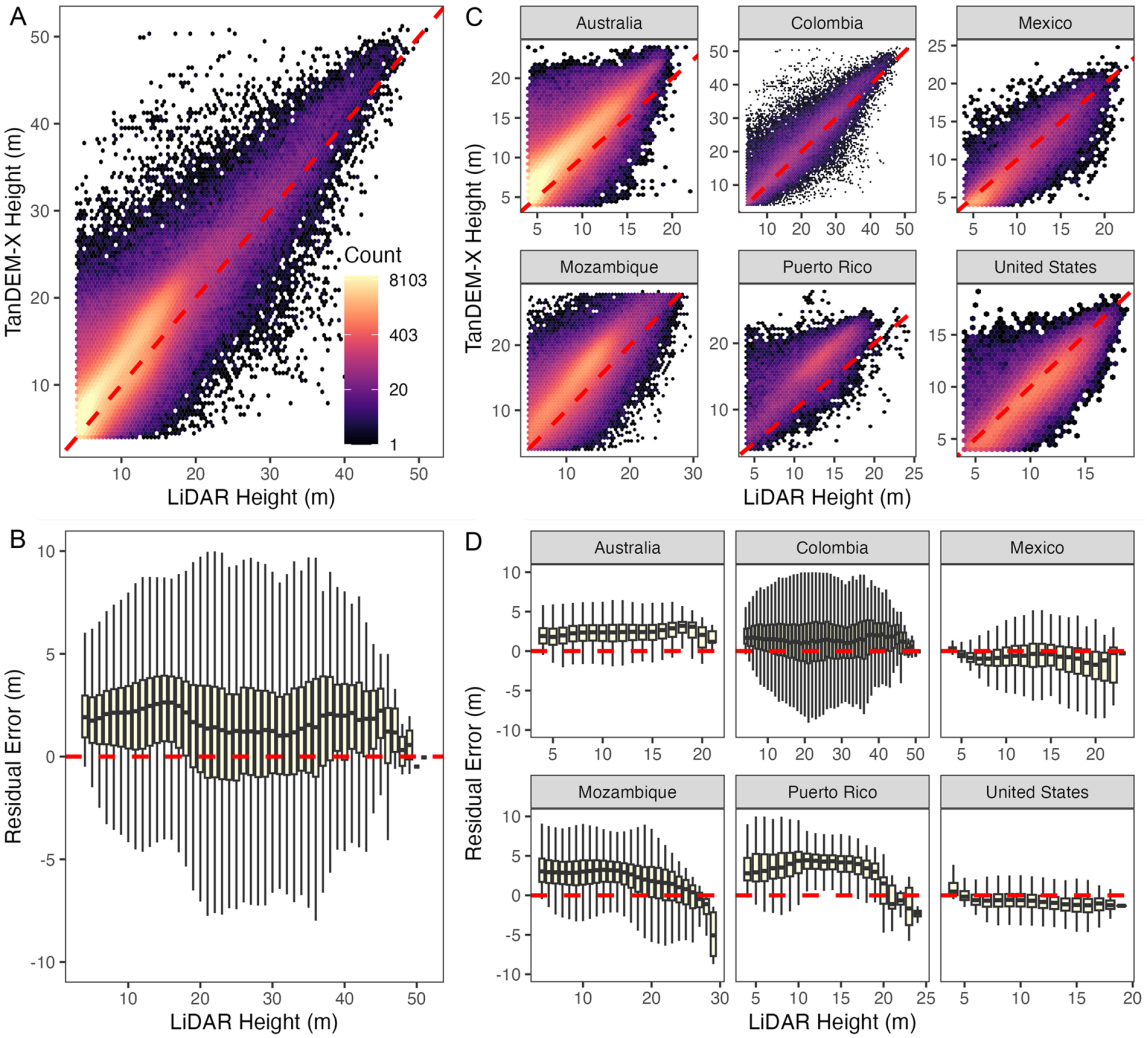
Validation of calibrated TanDEM-X height estimates using airborne LiDAR (ALS) data. Validation based on all sites (**A,B**) and from 6 locations (**C,D**), with one-to-one (top) and residual error (bottom) plots shown. The aggregate datasets show a positive bias (~2-4 m) in the TanDEM-X height estimates in the 10–20 m height interval compared to the ALS heights, with all other heights being relatively unbiased. Australia, Mozambique, and Puerto Rico show an error trend that increases with height, while other sites were nearly unbiased.

We aggregated the 1-meter canopy height model (CHM) maps to the same pixel resolution as the TDX data (~12 m, depending on latitude) using the mean. The resulting CHM raster is aligned to the same pixel grid as the TanDEM-X height map and approximates the per-pixel mean canopy height. One-to-one plots and residual error plots binned by 1-meter height intervals were created for visual interpretation of the errors with respect to TanDEM-X height estimates (Fig. 9). The final map had an overall RMSE of 2.4 meters globally when compared to the ALS data (Table 5).

**Table 5:** Airborne Lidar system (ALS) validation statistics.

site	Mean height	MAE	RMSE	bias
Australia	3.33	1.91	2.40	1.74
Colombia	16.65	3.44	5.34	1.16
Mexico	4.31	1.37	1.67	−1.16
Mozambique	9.17	2.85	3.67	2.34
PuertoRico	9.93	3.36	4.22	3.15
United States	8.20	1.64	2.07	−1.18
Overall	4.53	2.02	2.64	1.53

### Comparison of TDX mangrove height map and global forest height products

**Fig. 10:**
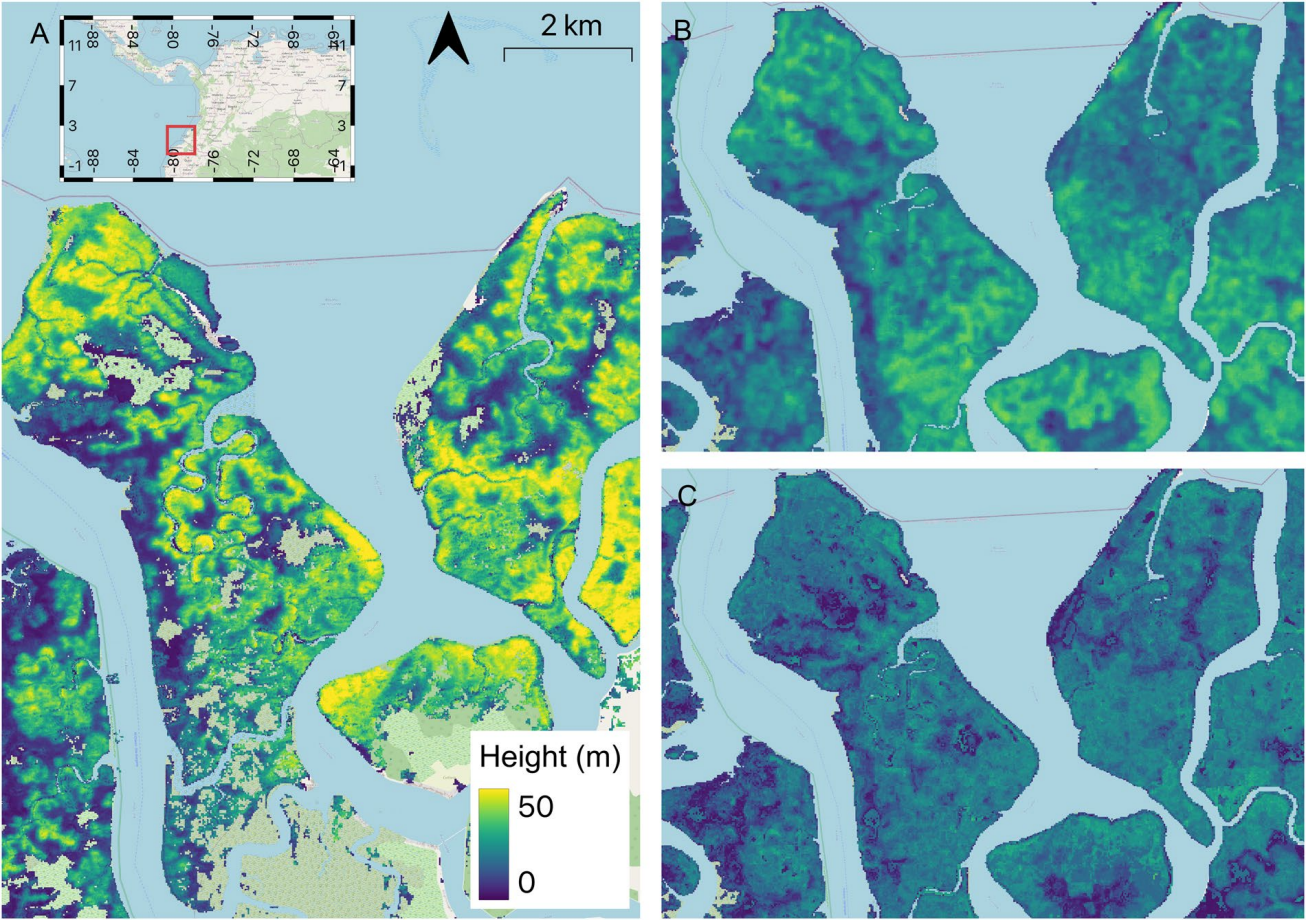
Comparison of mangrove canopy height products. (**A**) TanDEM-X (**B**) ETH (**C**) GLAD height products.

The new TanDEM-X mangrove canopy height (MCH_TX_) map provides a finer spatial resolution, greater height accuracy and a wider range of heights than other available high-resolution maps. However, there are significant differences with other existing height products, namely SRTM-derived mangrove canopy height map MCH_SRTM_^4^, Eidgenössische Technische Hochschule (ETH) global Sentinel-2 10 m canopy height^21^ and Global Land Analysis and Discovery (GLAD) height data^11^. First, the MCH_TX_ and MCH_SRTM_ are obtained from the radar-derived DEMs while the ETH and GLAD products are derived using model regression between Lidar-derived canopy height and optical remote sensing datasets. Since it was derived from Sentinel-2 data, the ETH product has a spatial resolution of 10 m. The GLAD product was derived with Landsat analysis-ready time-series with a coarser spatial resolution of 30 m (see Fig. 10B). Comparison with ETH and GLAD models highlights the quality of details and range of heights observed in MCH_TX_ (Fig. 10A). The direct elevation measurements from radar capture a much broader range of canopy heights with finer spatial details.

**Fig. 11:**
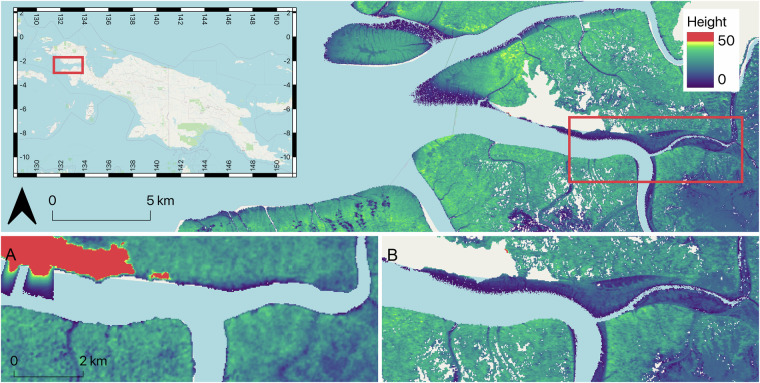
Examples of canopy height of MCH_TX_ over Bintuni Bay, West Papua. Red rectangle shows insets for (**A**) MCH_SRTM_ and (**B**) MCH_TX_.

The radar-derived maps represent the status of mangrove height in 2000 (MCH_SRTM_) and the period ~2011–2013 (MCH_TX_), and were generated using different mangrove extent products, respectively Giri *et al*.^22^ and Bunting *et al*.^6^. In Fig. 11, we show an example of differences observed in Bintuni Bay, West Papua. First on the Northwestern side of the insets A and B, an area that is not classified as mangroves in the GMW map (seen as a white patch) is misidentified as mangroves with unrealistic heights (red patch, inset A). Between 2000 and ~2012, the mangrove forest expanded into the river which explains the changes observed between MCH_SRTM_ (Fig. 11A) and MCH_TX_ (Fig. 11B).

## Usage Notes

The maps of mangrove height can be used to support evaluation of a variety of mangrove ecosystem services such as blue carbon stocks, coastal protection and value as a commodity, as well as improve our understanding of the role of mangroves as a climate mitigation and adaptation solution^23^. In addition, this new map can be used to assess sensor vertical resolution, uncertainty and geolocation^24^. Users should be aware of significant differences in mangrove extent and height accuracy when combining this new TanDEM-X-derived mangrove height with the 2000 SRTM-derived map^4^ to evaluate height. Finally, as evidenced by Table 3, users should validate the mangrove extent^25^ before reaching conclusions, particularly in island nations that exhibit extreme regional and local height values.

## Data Availability

The codes used to generate the datasets are available at: https://github.com/nmt28/TDX_Mangrove_Height. The new TanDEM-X mangrove canopy height as well as the GEDI data used for calibration and validation can be downloaded at the Oakridge National Laboratories’ Distributed Active Archive Centers (ORNL-DAAC)^20^.
